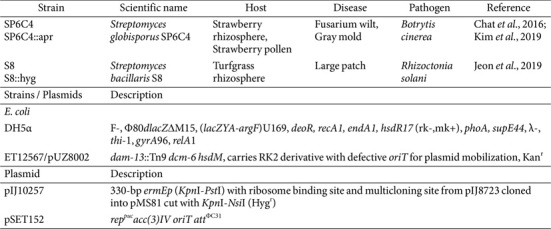# Root Rot Disease Biocontrol and Microbiome Community Modulation by *Streptomyces* Strains in Soybean

**DOI:** 10.4014/jmb.2502.02010

**Published:** 2025-05-26

**Authors:** Da-Ran Kim, Youn Min Ko, Donggyu Lee, Youn-Sig Kwak

**Affiliations:** 1 National Institute of Horticultural and Herbal Science, Rural Development Administration, Wanju 55365, Republic of Korea; 2 Division of Applied Life Science (BK 21 plus), Gyeongsang National University, Jinju 52828, Republic of Korea; 3 Institure of Agriculture and Life Science, Gyeongsang National University, Jinju 58282, Republic of Korea

**Keywords:** Beneficial microorganism, microbial community, soybean root rot, *Streptomyces*

## Abstract

Traditionally, phytopathogenic fungi control strategies rely primarily upon chemical fungicides, but fungicide resistance pathogen strains have appeared in the fields. Therefore, biocontrol approaches highlighted with sustainable agriculture aspects, especially the genus *Streptomyces*, are known to suppress numerous plant diseases. *Streptomyces bacillaris* S8 was isolated from turfgrass rhizosphere, and *Streptomyces globisporus* SP6C4 was obtained from strawberry pollen. Both strains showed excellent antifungal and antibacterial activities and suppressed various plant diseases *in vitro*. However, beneficial microorganisms are rarely studied and introduced to another effect on microbial communities when incompatible with the host. The present study aims to assess the potential of effective control of plant diseases by both strains in new crops and to assess the impact of endogenous microbiota. Various diseases pose significant concerns in soybean production, leading to substantial grain yield and quality losses. Root rot caused by *Fusarium* spp. is known to be the most problematic disease in the soybean cropping system. In the results, *S. globisporus* SP6C4 and *S. bacillaris* S8 showed antifungal activity against soybean root rot pathogen, but strain S8 had less than SP6C4. The strain SP6C4 played a role as hub-taxa in the early stage, and the strain S8 was a modulator in microbial communities. Our results demonstrate the antifungal activity of *S. globisporus* SP6C4 and *S. bacillaris* S8, which can be expected to grow and reduce the disease of soybeans. The S8 and SP6C4 can also modify the plant microbiota which may open a new dimension of crop microbiome research.

## Introduction

Soybean (*Glycine max*), a significant crop worldwide, is in increasing demand, but it has decreased yield per year due to various diseases [1-3]. Soybean root rot by *Fusarium* spp. caused serious problems for the production and sustainable cropping of the host plant [4-6]. Various species of the *Fusarium* genus, including *Fusarium oxysporum* f. sp. *tracheiphilum*, *F. oxysporum* f. sp. *vasinfectum*, and *F. solani* f. sp. *glycines* are known to be causal pathogens in the soybean root rot disease [7]. The root rot disease presents symptoms such as chlorosis of the leaf veins, premature of the leaf, wilting and brown to black in the roots, and eventually death [8].

Chemical fungicides have generally controlled the soybean phytopathogenic fungi, but fungicide-resistance pathogen strains have appeared in the soybean fields [9]. Therefore, biocontrol approaches are highlighted with the aspect of sustainable agriculture, especially the genus of *Streptomyces*, which is known to produce various antibiotic compounds as secondary metabolites [10]. As antibiotics for *Streptomyces* can suppress the diseases of plants, they attract attention as an object of abiotic stress agents [11]. *Streptomyces bacillaris* S8 was reported as a reliable control agent against brown patch disease in turfgrass [12-14]. Valinomycin was a key antifungal metabolite in the S8, known to make a nonribosomal peptide (NRPS) [14]. *S. globisporus* SP6C4 produces key antifungal peptides such as conprimycin and grisin, which have gray mold and *Fusarium* wilt disease suppressiveness, to control fungi that cause diseases in strawberries [15, 16].

Beneficial microorganisms were identified through microbial community analysis, which was normally performed below ground and above ground. Based on various research, key microbial groups, such as Actinomycetota, Pseudomonadota, and Bacillota, were highlighted [15, 17]. Belowground environments, such as the rhizosphere and root endosphere, harbor diverse beneficial microbes that help protect the host from soilborne pathogens [18, 19]. Additionally, the enriched beneficial microorganisms can alter the microbial community structure, making it more favorable and healthier for the host [19-22]. In this study, we focused on the effects of suppressing diseases in below-ground areas of the plants. We selected the *S. bacillaris* S8 and the *S. globisporus* SP6C4 to control soybean diseases. This study evaluated the antifungal activity of strain S8 and strain SP6C4 against *F. oxysporum* and the applicable alternative host, such as soybean. Additionally, we presented the endogenous microbial community response to introduce the biological control agents. Lastly, we expect that the SP6C4 and S8 strains may be useful in agriculture, and the findings could be useful to control soybean diseases.

## Materials and Methods

### Plant Growth Condition

The soybean (cv. Daewon) seed was sterilized with 1% NaOCl for 10 min and washed twice using 50 ml of ddH_2_O. The seeds were placed on filter paper (diam. 8 cm) and dried for 2 h at room temperature, which were transferred to sterilized cotton balls. Each cotton ball was put on the petri dish and germinated for 3~5 days in dark conditions at 28°C. After the germination, plant a plastic square pot (diam. 15 cm) with 600 g of raw soil (collected from the university’s rice paddy field) and grow in the glass house. The growth condition was 8 h dark/21°C ± 3 and 12 h light/32°C ± 3, relative humidity was 60%~75%, and the plant grown for 6 weeks.

### Bacteria Culture Condition

For confrontation assay, two bacteria strains (*S. bacillaris* strain S8 and *S. globisporus* strain SP6C4) were grown on MS media (20 g of mannitol, 20 g of soya flour per L) and cultured at 28°C for 5 days (Table 1). The spores were collected using a sterilized cotton ball and a 15-ml syringe for filtration, calculated at an OD_600nm_ of 0.7 in a Microplate Reader (BioTek, Synergy H1, Nederland). Then, the spore stock was inoculated on a filter disk (0.8 diameter) in PDK agar media (10 g of potato dextrose broth, 10 g of peptone, 20 g of agar per L), and it was incubated at 28°C for 3 days for sporulation and added fungal pathogen at the center of the plate. *Fusarium oxysporum* Funbio 41 (KACC no. 410745) was cultured on PDA (24 g of potato dextrose broth, 20 g of agar per L) at 28°C for 5 days. The assay was performed for 7 days at 28°C.

For biocontrol and colonization assay, *S. bacillaris* strain S8::hyg [13] and *S. globisporus* strain SP6C4::apr [23] were streaked on MS media (hygromycin, 80 μg/ml and apramycin, 50 μg/ml) and incubated at 28°C for 5 days. The single colony was inoculated on 5 ml of PDK broth medium (10 g of potato dextrose broth, 10 g of peptone per L), which was placed at 28°C for 5 days at 250 rpm (Table 1). The seed culture was added to 500 ml of PDK broth and incubated at 28°C for 10 days with 150 rpm, and the cultured bacterium was separated into 50 ml of falcon tube and centrifugation at 25,910 ×*g* for 20 min for discarded nutrient medium. After the centrifugation, a supernatant and then 50 ml of ddH_2_O were gently pipetted to the washed nutrient medium. The wash step was repeated twice, and finally, 50 ml of ddH_2_O in a 50-ml falcon tube and 1 ml of bacteria cells were put on the 9 ml of ddH_2_O; it was serial diluted up to 10-7, and 100 μl was spread on PDK agar medium. The plate was incubated at 28°C for 5 days and evaluated Log colony forming unit (CFU) per ml.

### Biocontrol and Colonization Assays

One-week-old soybeans were washed with sterilized water and then placed in distilled water. Bacteria cells were prepared at a concentration of 10^6^ CFU/ml with 0.1% carboxyl-methylcellulose (CMC), and three plants were dipped in 150 ml of the bacteria mixture for 10 min. At the dipping step, the mixture was briefly stirred in each 3 min, and then the plants were replaced in a square plastic pot (15 cm × 15 cm) with 600 g of low soil (*n* = 9). For root rot diseases, indexing from 0 to 4 was used to assess the presence of root necrosis (0: no symptoms; 1: necrosis < 20%; 2: necrosis 21-50%; 3: necrosis > 51%; 4: plant death). Each treatment group's disease index (DI) was measured and statistically analyzed using the Kruskal-Wallis test in the R package [24].

The soybean plants were sampled at 2 weeks, 3 weeks, 4 weeks, and 5 weeks, then cut off to root tissue, sterilized surface using 70% EtOH for 1 min 30 sec, 1% NaOCl 1 min 30 sec, and washed ddH_2_O with 3 times. The tissues were put in the 50 ml falcon tube with 30 ml of 2.5 mM 2-(N-morpholino)ethanesulfonic acid (MES) buffer [17]. The tubes were placed in a sonication bath at 10°C for 20 min, and tissues were placed on filter paper (diam. 15 cm) on a clean bench for 2 h. Finally, 1 g of root tissues were homogenized at mortar with 9 ml of 2.5 mM MES buffer, which was filtered with a 2-layer of miracloth (Merck Millipore, Germany). A completely homogenized root extract (1 ml) and 1 g of rhizosphere mixed with 9 ml of ddH_2_O in each 15 ml tube and diluted up to 10^-7^ using serial dilution. Colonization density of strains was detected at PDK + hygromycin (final concentration: 80 μg/ml) for S8 and at PDK + apramycin (final concentration: 50 μg/ml) for SP6C4.

### Microbial Community Analysis

Soybean sampling consisted of two different tissues, which were the rhizosphere and the root endosphere. Rhizosphere was collected from the root surface, and the root tissue was sterilized with 70% EtOH for 1 min and 1% NaOCl for 1 min; it was washed ddH_2_O more than five times to obtain root endosphere. The root tissues were dipped in 2.5 mM of MES buffer (pH 5.7) and sonicated for detached surface microorganisms and dust. The tissues were dried on 15 cm diam. filter paper for 1 h, then cut off the 2 cm by soil line and root tip; the section might be contaminated with other solution; the root tissue was transferred to a cleaned 50-ml tube, and 2.5 mM MES buffer was in each g/ml. A mixture was ground to extract the endophyte microbes from the buffers. The buffer (500 μl) was transferred to Lysing matrix E-tube FastDNA SPIN Kit (MP Biomedicals, USA) with 122 μl of MT buffer, 978 of μl of sodium phosphate buffer, and homogenized with FastPrep-24TM Classic bead homogenizer (MP Biomedicals) following the manufacturer's instructions. Total DNA was washed with 500 μl of SEWS-M buffer with EtOH and centrifuged at 14,000 ×*g* for 1 min. The buffer was discarded and replaced with the catch tube on the membrane tube, and 50 μl of DES buffer was added. The DNA quality was calculated with a spectrometer (Nanodrop 2000C, Thermo Fisher Scientific, USA). Before an endophyte microbial illumine sequencing, it was necessary to block the PCR stem to block plant plastid DNA amplicon chimera [25]. At the blocking PCR performed with 20 μl of KAPA HiFi HotStart ReadyMix (Roche, Switzerland), PNA (5’-GGCTCAACCCTGGACAG-3’), mPNA (5’-GGCAAGTGTTCTTCGGA-3’), 515F primer (5’ TCGTCGGCAGCGTCAGATGTGTATAAGA GACAGGTGCCAGCMGCCGCGGAA-3’), and 805R primer (5’-GTCTCGTGGGCTCGGAGATGTGTATAA GAGACAGGACTACHVGGGTATCTAATCC-3’). The blocking PCR conditions were as follows: initial denaturation at 98°C for 5 min, followed by 22 cycling steps consisting of denaturation at 98°C for 1 min, PNA at 78°C for 10 sec, annealing at 55°C for 30 sec, extension at 72°C for 1 min, and final extension at 72°C for 5 min [24]. After the PCR fragment was amplified in the 16S rRNA partially V4 region and purified with an SV kit (GeneAll, Republic of Korea). The product is used at pyrosequencing with Illumine Miseq (2 × 300 bp) by Macrogen (Republic of Korea).

The removed barcode sequences and quality check were performed with fastq format, and forward length of 240-bp and reverse length of 220-bp were merged to amplicon sequence variants (ASVs). The merged sequences were clustered by DADA2 (version 1.20) in R (version 4.3.3), as described by Callahan *et al*. [26]. Taxonomic classifications were carried out utilizing the IDTAXA classifier from the DECIPHER package (Version 2.20), employing the SILVA (v138.1) SSU database for each ASV, following the methodology outlined by Murali *et al*.[27]. In the bacterial community, analysis was implemented for alpha diversity, bar plots of bacterial proportion, principal coordinates analysis (PCoA), and Zi-Pi plots using the phyloseq package (version 1.36) and microeco package (version 1.9.0). Results were visualized using ggplot2 (version 4.3.3) in R.

### Statistical Analysis

Statistical analyses were performed using Welch’s *t*-tests and linear regression ANOVA. In cases where the data did not exhibit normality or equal variance, Kruskal-Wallis tests were applied. Post-hoc comparisons were conducted using Tukey’s HSD. For PCoA, pairwise Adonis was used to analyze the data. Graphs were generated using the ggplot2 package in R.

## Results

### SP6C4 and S8 Showed Substantial Antifungal and Effective Colonization Activity on Soybean

**Fig. 1:**
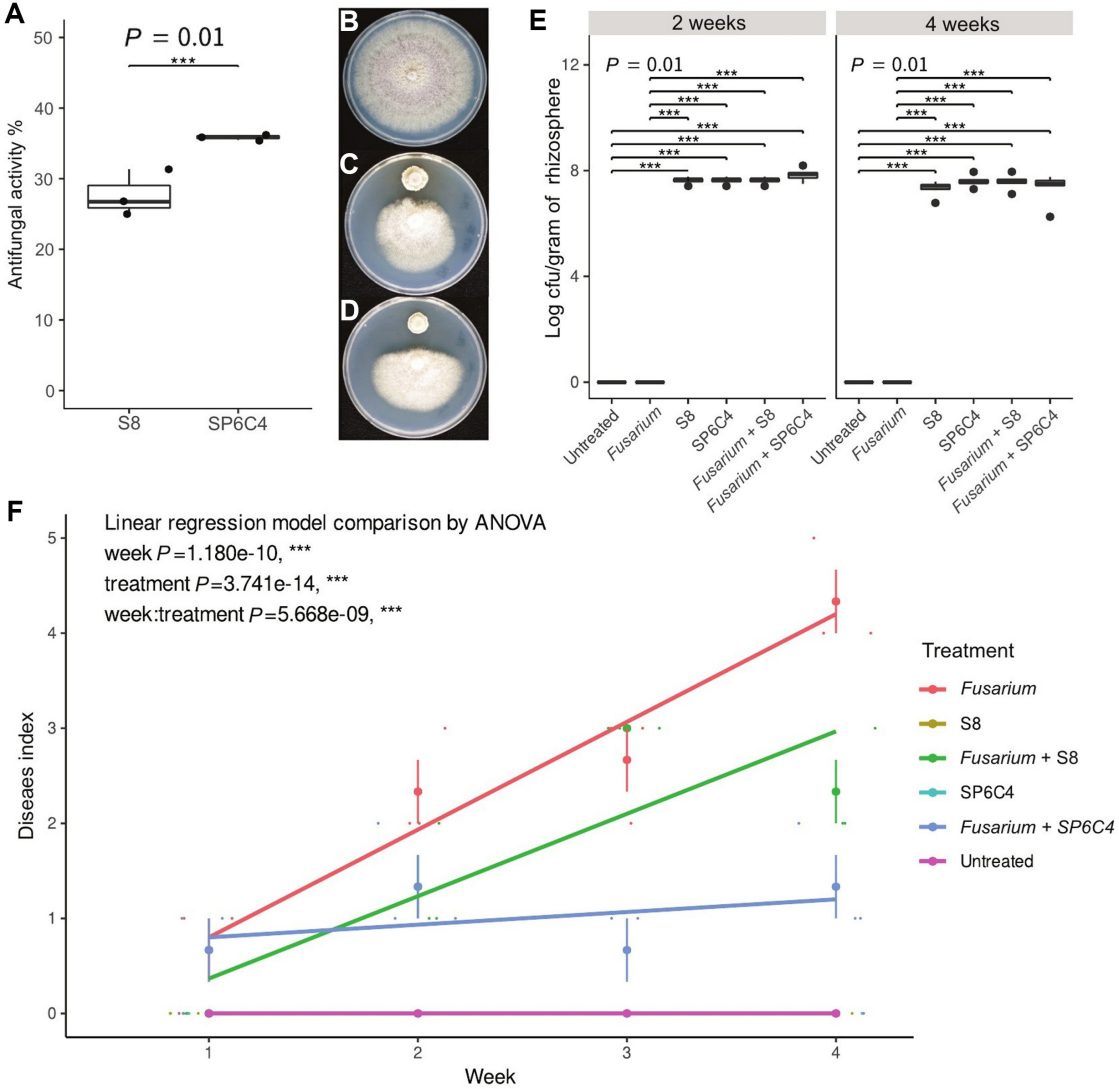
Antifungal activity and soybean root rot disease suppression by *S. globisporus* SP6C4 and *S. bacillaris* S8. (**A–D**) The bacterial suspensions (10^6^ CFU/ml) were dotted on 0.8 cm diam. filter disk using the disk diffusion method. After 3 days, *Fusarium oxysporum* agar plug (0.4 cm diam.) was added to the disk. The bacterial suspensions were preinoculated with 20 μl of OD_600_ 0.6 (*n* = 3). (**E**) Evaluation of colonization ability of the strains on soybean rhizosphere. The cultured bacterial cells (10^6^ CFU/ml) were mixed with 0.1% CMC and dipped at the root. The plant was grown in greenhouse conditions. Below-ground disease (*F. oxysporum* 10^5^ chlamydospore/ml) inoculated with drenched in each plant by 10 ml (plastic pot, 9 cm diam.). Colonization was measured by serial diluting, rhizosphere from 10^-1^ to 10^-7^ and Tukey’s honestly significant difference test, box plots were calculated with ggplot2 package of the R (4.0.3) significance for *p* < 0.05 (mean ± SD). (**F**) Biological control effect by *Streptomyces bacillaris* S8 and *Streptomyces globisporus* SP6C4 against *F. oxysporum* in the glass greenhouse. The disease index is based on the percentage of leaf area affected by necrosis. Disease index is the percentage of the browning and rotting roots (*n* = 5). The *Fusarium* sp. (10^5^ CFU/ml) was drenched after the bacteria was treated. They were grown in the glass greenhouse for 28 days, and the temperature was from 28°C to 30°C, with 70-80% relative humidity. The experiment was conducted with three replicates, and statistical analysis was performed using a linear regression model calculated using ANOVA and the ggplot2 package of the R (4.0.3).

**Fig. 2:**
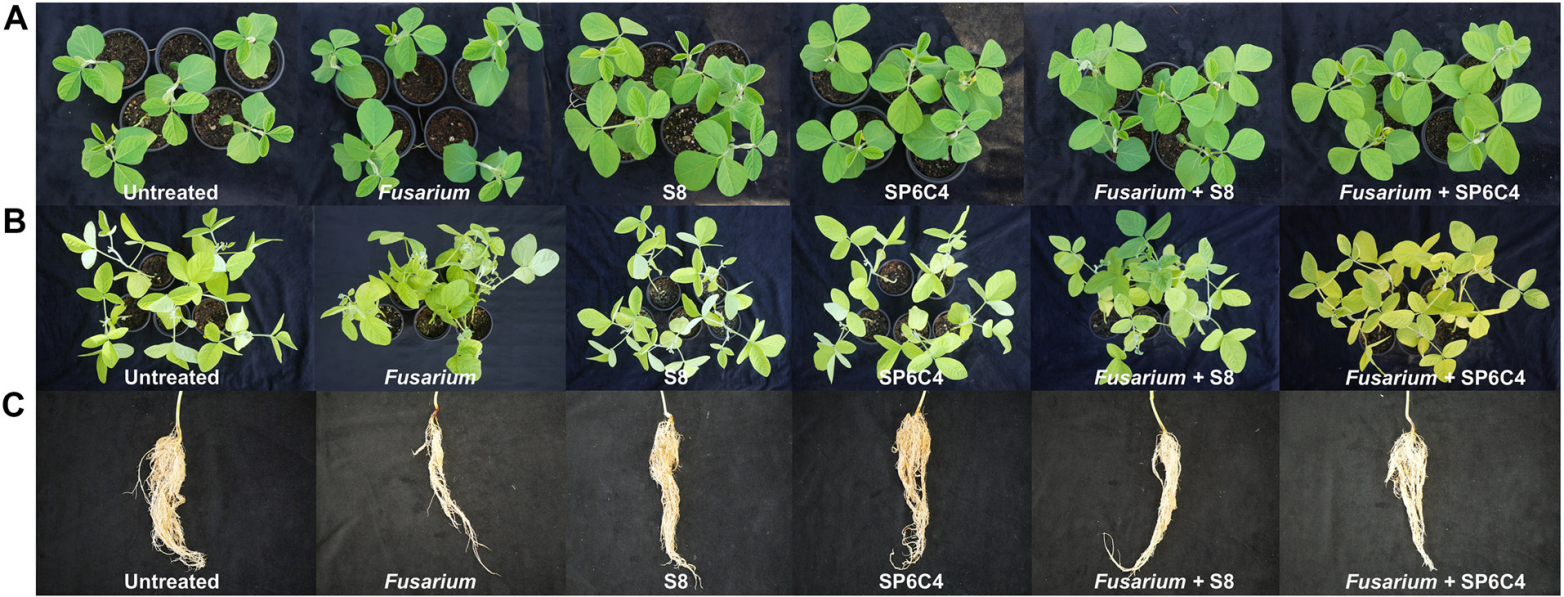
Symptoms of root rot disease of soybean and suppression of the disease occurrence by *Streptomyces* strains. Soybean plants were cultivated in a glass greenhouse under 12 h light and a temperature of 32°C ± 3; 8 h dark and 21°C ± 3. Before the pathogen treatment, inoculation using *S. bacillaris* hygromycin resistance type (strain S8) and *S. globisporus* apramycin resistance type (strain SP6C4). The below-ground pathogens were employed as chlamydospore stock (10^5^ spore/ml). (**A**) 2 weeks; (**B**) 4 weeks; (**C**) at 4 weeks root section.

Each *Streptomyces* strain was inoculated on PDK media with *F. oxysporum* to assess antifungal activities, which resulted in reduced fungal growth. SP6C4 demonstrated greater antifungal activity (37.6%) compared to S8 (26.8%) (Fig. 1A and 1B-1D). In the colonization assay, the bacteria had properties in the rhizosphere up to 108 CFU/g until 4 weeks (Fig. 1E), but at disease control outcomes differed between the S8 + *Fusarium* and SP6C4+ *Fusarium* treatments (Fig. 1F). After 4 weeks, untreated, S8-only treatment and SP6C4-only treatment showed no disease symptoms. The SP6C4 + *Fusarium* treatment had a disease index level of 1, while the S8 + *Fusarium* treatment had a disease index level of 2.7, and the *Fusarium*-only treatment had a disease index level of 4 (Fig. 1F). All treatments were grouped into four statistically significant categories (treatment *P* = 3.714e-14, ***; week *P* = 1.180e-10, ***), no disease symptom (untreated, S8-only treatment and SP6C4-only treatment), SP6C4 + *Fusarium*, S8 + *Fusarium*, and *Fusarium*-only treatment (Fig. 1 F). Samples from the rhizosphere and root endosphere (*n* = 5) and the root section were damaged by *Fusarium* root rot disease; *Fusarium*-only treatment displayed darkened tissue and reduced root hair, which contrasted with the healthier root sections observed in other treatments (Fig. 2).

### SP6C4 and S8 Have Different Effects on Disease Control and Microbial Community

**Fig. 3:**
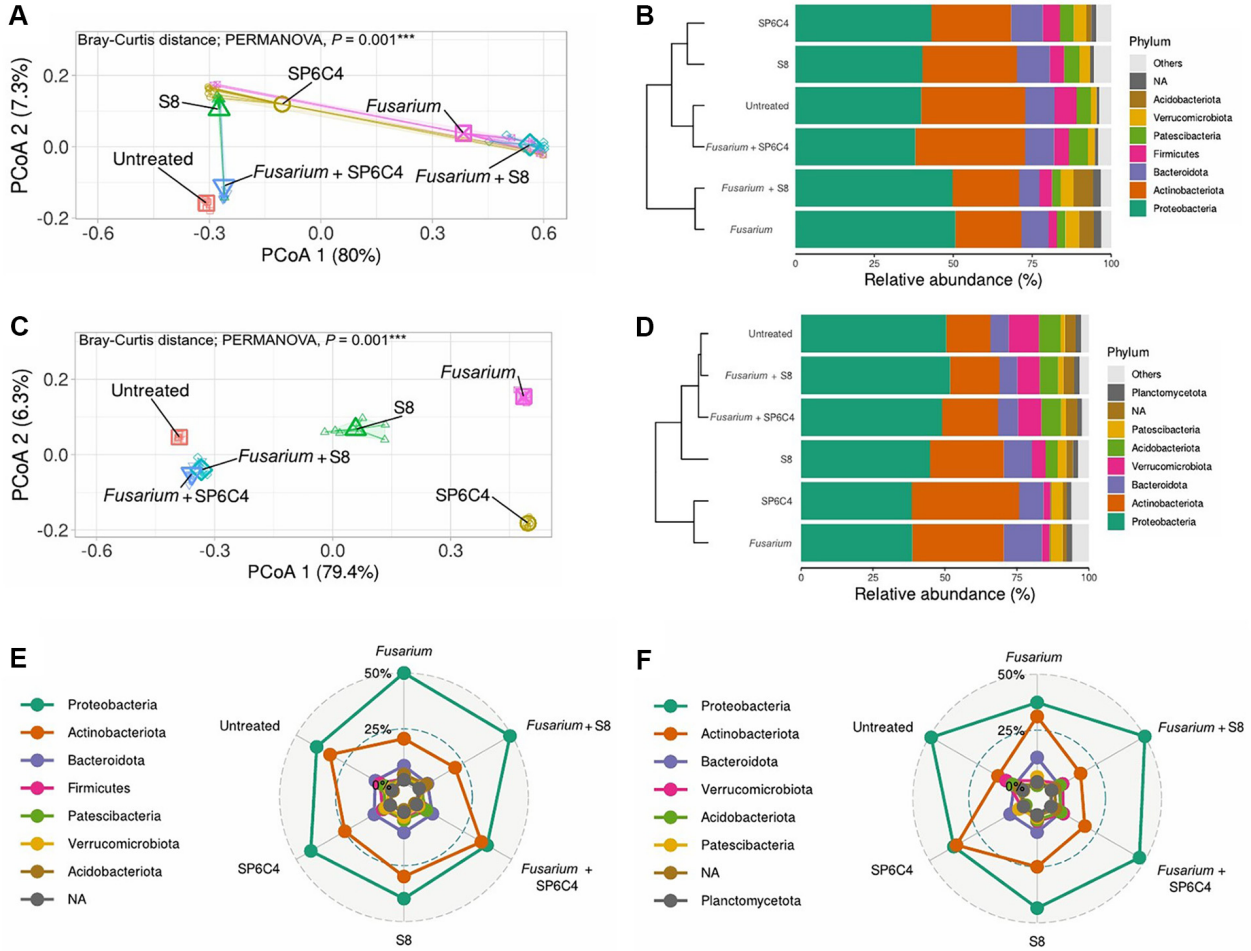
Compositions of rhizosphere microbial communities in varieties plots. (**A**) for 2 weeeks and (**C**) for 4 weeeks, Bray-Curtis distance of ASVs relative abundance based on β-diversity analysis. The classical principal coordinate analysis (PCoA) is used to represent the Bray- Curtis distance in a graph. (**B**) for 2 weeks and (**D**) for 4 weeks, Stacked bar plots display the average proportions of bacterial populations, and microbial compositions are displayed on the right. Based on Bray- Curtis distance metrics, the clustering dendrogram of the microbial communities and hierarchical clustering dendrogram is shown on the left. (**E**) for 2 weeks and (**F**) for 4 weeks: The radar graph shows the relative abundance of rhizosphere microbial richness conversion of treatment groups.

The SP6C4 strain presented a great advantage in being able to control soybean root rot pathogen, and the strain S8 had less disease suppresses effect but was well-colonized at the rhizosphere. These results raise two important questions: First, can the SP6C4 strain protect against *F. oxysporum* in soybeans? Second, could the reduced disease suppression by the well-colonized S8 strain that the endogenous microbiota indicates is modulating the plant's health? To explore these questions, we collected samples from the rhizosphere and root endosphere for 2 and 4 weeks, with each treatment replicating 9 samples. The NCBI accession numbers are listed in Tables S1 and S2. In the first step, sequencing quality was calculated using a rarefaction curve and coverage data (Fig. S1 and Table S3). We investigated microbiota modulation in soybeans. At 2 weeks, the *Fusarium*-only treatment rhizosphere and *Fusarium* + S8 showed similar taxa in the rhizosphere, particularly from the Acidobacteriota. However, in the root endosphere, the *Fusarium* + S8 treatment displayed a significantly higher proportion of Burkholderiaceae (over 30%) compared to the *Fusarium*-only treatment (Fig. S3A and S3B). At 4 weeks, the *Fusarium* + S8, *Fusarium* + SP6C4, S8, and untreated treatments resembled the microbial community with a higher Acidobacteriota group than *Fusarium*-only treatment and SP6C4-treatment (Fig. S2C). While in the root endosphere, the S8-only treatment exhibited distinct taxa with elevated levels of *Paenibacillaceae* (15.3%~25.3%), *Streptosporangiaceae* (6.7~27.3%) and *Thermomonosporaceae* (6.3%~31.2%) contrast other treatments, that had many *Nocardiaceae* (Fig. S2D). Additionally, the Actinobacteria phylum was prominent in the untreated and *Fusarium* + SP6C4 treatments, which shared a similar microbial community structure in the dendrogram at 2 weeks, whereas the S8-only and SP6C4-only treatments were distinct from the untreated but still showed a resemblance to each other at 2 weeks (Fig. 3A, 3B, and 3E). At 4 weeks, the microbial community of untreated, *Fusarium* + S8 and *Fusarium* + SP6C4, which had grouped. But S8-only treatment, SP6C4-only, and *Fusarium*-only treatments were districted to each microbial community (Fig. 3C and 3D). The S8 treatment recruited its own specific community, characterized by more than 25% of Proteobacteria that distinguished it from both the *Fusarium*-only and SP6C4-only treatments at 4 weeks (Fig. 3F). In contrast, the root endosphere exhibited a delayed microbial community response to both beneficial microorganisms and the pathogen, except for the *Fusarium* + SP6C4 treatment at 2 weeks (Fig. S3A and S3B). At 4 weeks, the *Fusarium* + S8 treatment resembles untreated and *Fusarium* + SP6C4 (Fig. S3C and 3D). Among the different beneficial microorganisms, SP6C4 was more effective in protecting against the plant pathogen because *Fusarium* + S8 and S8-only treatments were not significant at 2 weeks, but at 4 weeks, two treatments were distinguished by statically on pairwise Adonis (Table S4 to S7).

**Fig. 4:**
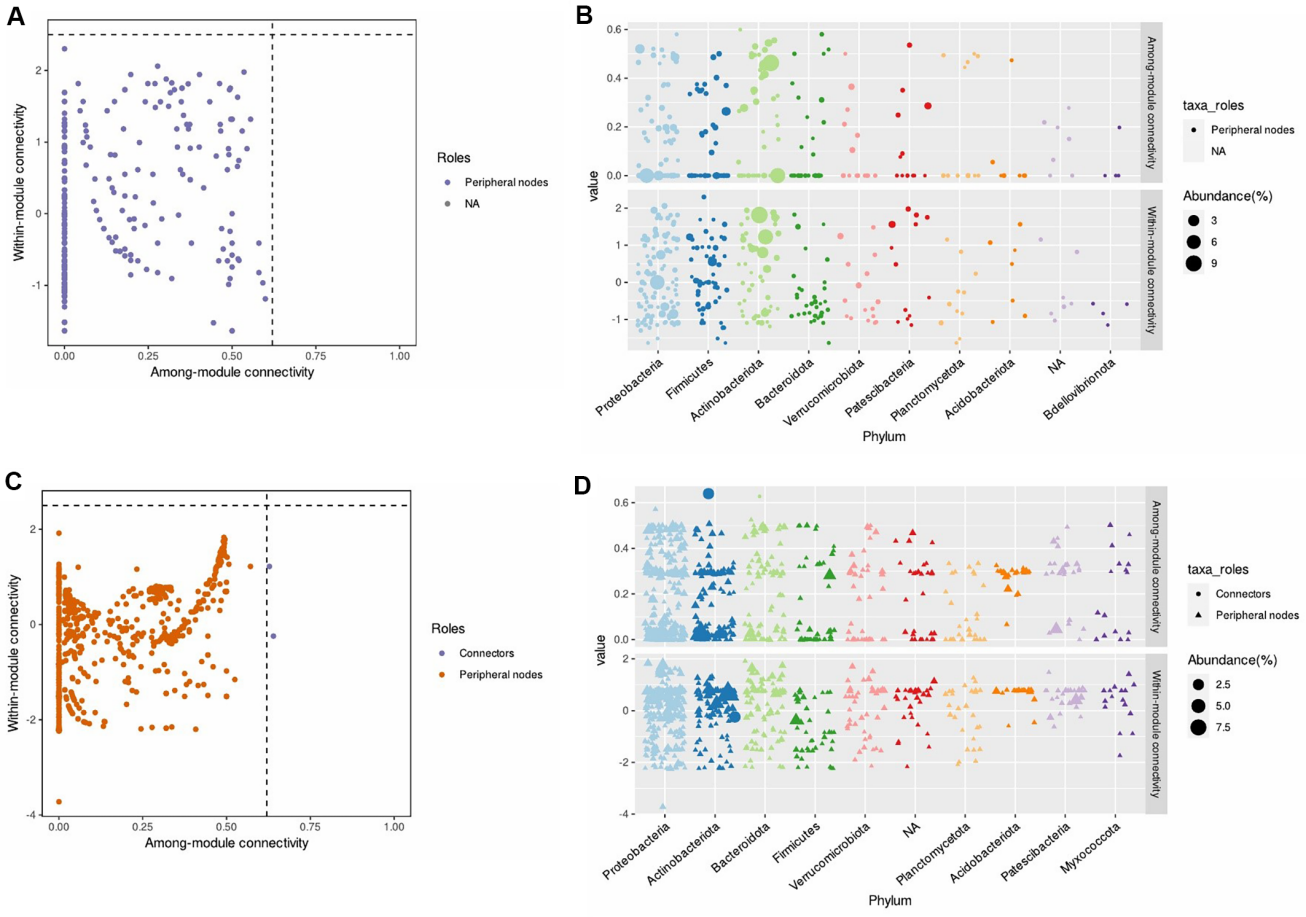
Zi-Pi plot of the rhizosphere microbial module at 2 weeks. (**A**) and (**B**) Zi-Pi plot based on the rhizosphere of only S8 and only SP6C4. (**C**) and (**D**) Zi-Pi plot based on the rhizosphere of Untreated and *Fusarium* + SP6C4. The symbol represents a connector (purple) for Actinobacteria. The topological role of each ASV was determined according to the scatter plot of within-module connectivity (Zi) and among-module connectivity (Pi). Based on WGCNA (Weighted Gene Coexpression Network Analysis), the Zi-Pi plot of rhizosphere bacteria illustrates threshold values for Zi and Pi. Module taxa ASVs are defined by Zi > 2.5 and/or Pi > 0.625. The Zi-Pi plot was performed with the microeco package (v.1.9.0) in R (v.4.3.0).

**Fig. 5:**
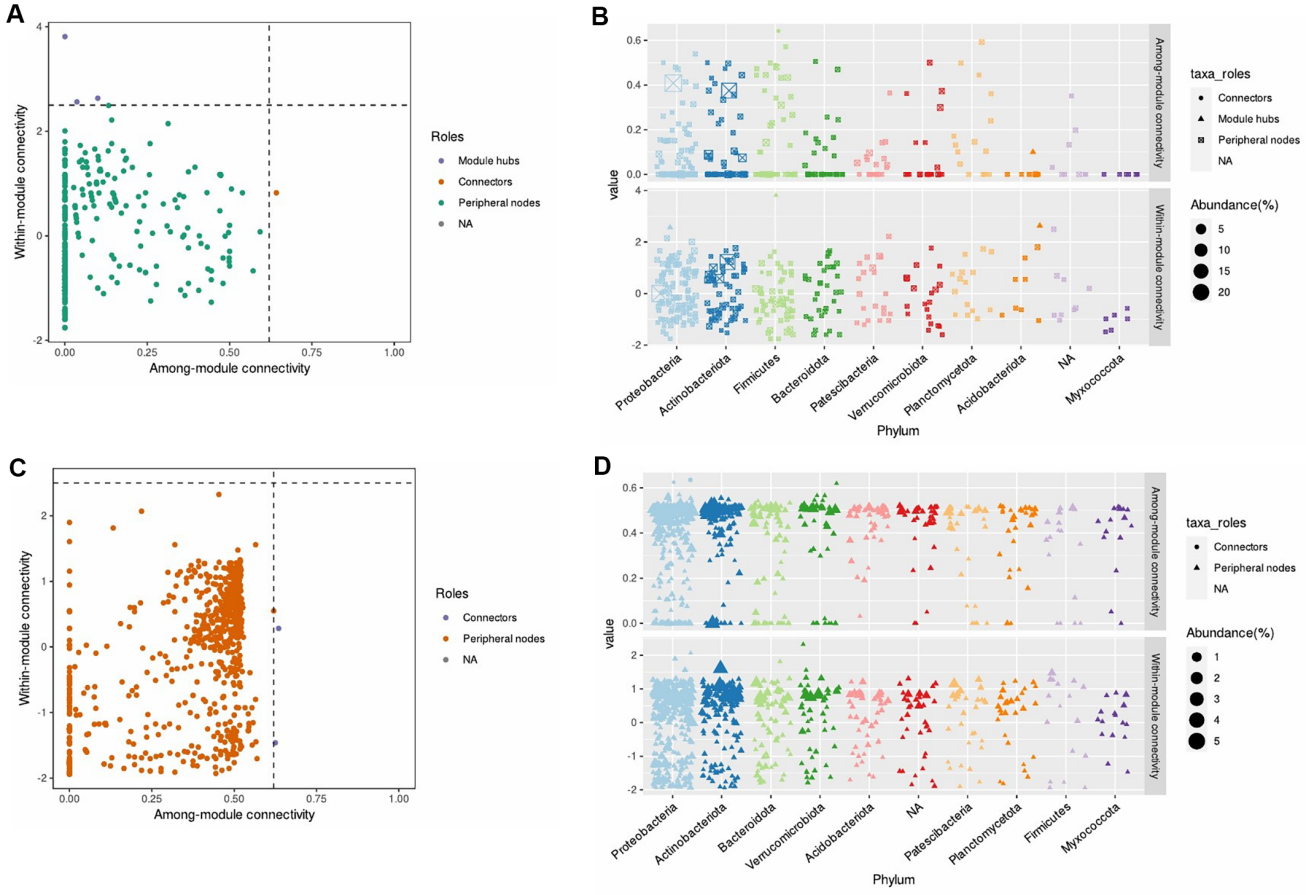
Introduced *Streptomyces* SP6C4 and S8 alter the microbial community at 4 weeks. (**A**) and (**B**) Zi-Pi plot based on the rhizosphere of Untreated, *Fusarium* + SP6C4 and *Fusarium* + S8. Each symbol shows a hub module (purple) for Firmicute, Proteobacteria, and Acidobacteriota and a connector (orange) for Firmicutes. (**C**) and (**D**) Zi-Pi plot based on the rhizosphere of only S8, which had connectors for Proteobacteria, not Actinobacteria. Within-module connectivity (Zi) and among-module connectivity (Pi) were determined as the topological role in ASVs at scatter plots. The WGCNA module connectivity had threshold values at Zi > 2.5 and/or Pi > 0.625, which are defined as module taxa ASV. The Zi-Pi plot was performed with the microeco package (v.1.9.0) in R (v.4.3.0).

After that, within-module (Zi) and among-module connectivity (Pi) plots with low disease incidence and *Streptomyces* (S8-only treatment and SP6C4-only treatment) inoculated rhizosphere have no module taxa (Fig. 4A and 4B) at 2 weeks. Otherwise, untreated and *Fusarium* + SP6C4 rhizosphere have an Actinobacteria (sq_6, genus: *Streptomyces*) identified as connectors (Fig. 4C and 4D). At 4 weeks, two different beneficial microorganisms were altering the endogenous microbial communities in soybean. *Fusarium* + S8 and *Fusarium* + SP6C4 rhizosphere were additive module microbes such as connector: Firmicutes; hub: Firmicute, Proteobacteria, and Acidobacteriota (Fig. 5A and 5B). In the S8-only treatment, the rhizosphere had acquired two connectors as Proteobacteria but not Actinobacteria (Fig. 5C and 5D).

## Discussion

Soybean, a major crop worldwide, has a decreased yearly yield due to many pathogens [1-3]. In a previous study, *Colletotrichum truncatum*, *Fusarium* spp., and *Phytophthora sojae* are known to be the most problematic pathogens in soybean. Therefore, those pathogens have been tried to control with conventional fungicides and alternative control methods [5, 28]. Nowadays, biocontrol has been highlighted in plant disease control with sustainable agriculture aspect, especially the *Streptomyces*, which has been integrative due to the bacterium producing many antibiotic compounds as secondary metabolites [10, 11]. To suppress the disease by using biocontrol way, considering the antifungal activity of *Streptomyces* is on the rise [9, 14, 16].

*S. bacillaris* S8 suppressed a large patch disease in turfgrass, and it was also nominated in the soybean health microbial community as a key microbe [12-14]. Also, newly had antifungal activities identified in non-host soybeans were protected from *Xanthomonas citri* pathogens by strain S8 with NRPS, such as bafilomycin and salinomycin [9]. Additionally, strain S8 was mentioned as a key taxa within the microbial community [21]. Another strain, *S. globisorus* SP6C4, had antifungal ability against *F. oxysporum* f. sp. *fragariae*, a causal pathogen of *Fusarium* wilt disease in strawberries, and it showed a different effect in reducing the fungal pathogen growth [15, 29]. In a previous study, the mycelial growth of *Botrytis cinerea*, *Colletotrichum gloesporioides*, *Phytophthora nicotine*, and *Rhizoctonia solani* was clearly inhibited by *Streptomyces* sp. S4-7 (genome identically 99.9% with *S. globisporus* SP6C4) [24]. We hypothesized that *Streptomyces* strains S8 and SP6C4 may show protection activity in non-host crops and other crop plants such as soybeans. Two beneficial microorganisms were not verified for the real activities of soybean pathogens, and in this study, the possibility of the two strains extending host plants and disease controlling was investigated on a wide spectrum against plant pathogens and diseases.

We conducted the experiment to observe the antifungal activity of *Streptomyces* strains S8 and SP6C4 on soybean fungal diseases that are currently caused by yield-reducing factors in soybeans. As a result, we verified that strain S8 and strain SP6C4 have antifungal activity on soybean root rot pathogen. Through these findings, we confined certain strains of *Streptomyces* that could be useful to control soybean fungal diseases, and it could be a broad understanding of the biological control of soybean-associated fungal pathogens. However, the S8 strain presented no significant advantage to be able to control the below-ground disease. Interestingly enough, S8 was colonized more than the number of 10^5^ CFU/ml on the rhizosphere. The results have a concern when two different beneficial microorganisms might be playing distinct roles within the microbial community.

We got the two points about the microbial communities, point 1 (SP6C4 vs SP6C4 + *Fusarium*, S8 vs S8 + *Fusarium*): Beneficial microorganisms are modulated microbiota when the presence of pathogens. These microorganisms become activated in the environment when pathogens are present, allowing them to perform their designated roles. That prediction suggests interactions between beneficial microorganisms and pathogens, where pre-existing beneficial microorganisms recognize and respond to pathogens, activating mechanisms that protect the host rather than being triggered by the host itself. Point 2 (Module change): The early stage of beneficial microorganisms alters the microbial community depending on the type of microbes, the distinct ways that *Fusarium* + SP6C4 treatment shifted to untreated at 2 weeks, and also, a shifted the hub-modules within the microbial communities were identified on Actinobacteria. In the case of S8-treated groups, both *Fusarium* + S8 and S8 alone showed more extensive hub-module modifications at 4 weeks. In particular, early-stage beneficial microorganisms act as a hub to suppress the plant disease, while later-stage beneficial microorganisms may modify the community’s module to sustain host resistance against plant pathogens.

Our findings show the importance of the early prefaced of beneficial microorganisms and their roles as modulators within ecosystems. Thus, it may be used to control a broad range of hosts within the microbial community. These findings are of particular interest when two different beneficial microorganisms fulfill distinct roles in the microbial community. Finally, we provide evidence of the interactions between beneficial microorganisms and pathogens, further emphasizing their ecological significance.

## Supplemental Materials

Supplementary data for this paper are available on-line only at http://jmb.or.kr.



## Figures and Tables

**Table 1:** Bacteria and plasmids used in this study.