# Supplementary material for: Root Rot Disease Biocontrol and Microbiome Community Modulation by Streptomyces Strains in Soybean
Source: J Microbiol Biotechnol. 2025 May 26;35:e2502010. doi: 10.4014/jmb.2502.02010 (PMC12149395; doi:10.4014/jmb.2502.02010)
Supplement: Supplementary file 1 [file jmb-35-e2502010-supple.pdf]

J. Microbiol. Biotechnol. 2025. 35: e2502010
https://doi.org/10.4014/jmb.2502.02010

Supplementary Tables and Figures

Root Rot Disease Biocontrol and Microbiome Community Modulation by *Streptomyces* strains in Soybean

Da-Ran Kim, Youn Min Ko, Donggyu Lee*, Youn-Sig Kwak*

Table S1. NCBI accession number list for rhizosphere samples.

Bioproject	Name	Biosample	Sample	accession	library_ID
PRJNA1156826	24-FunBio-FO_Ro_SP6C4_S8-31July	SAMN43501980	Rhizosphere	SRR30554031	H_2000_1
				SRR30554032	H_2001_1
				SRR30554026	H_2002_1
				SRR30554015	H_2003_1
				SRR30553975	H_2004_1
				SRR30553964	H_2005_1
				SRR30553951	H_2006_1
				SRR30554004	H_2007_1
				SRR30553940	H_2008_1
				SRR30553983	H_2010_1
				SRR30554033	H_2011_1
				SRR30554042	H_2012_1
				SRR30554043	H_2013_1
				SRR30554044	H_2014_1
				SRR30554045	H_2015_1
				SRR30554046	H_2016_1
				SRR30554022	H_2017_1
				SRR30554023	H_2018_1
				SRR30554024	H_2060_1
				SRR30554025	H_2061_1
				SRR30554027	H_2062_1
				SRR30554028	H_2063_1
				SRR30554029	H_2064_1
				SRR30554030	H_2065_1
				SRR30554021	H_2066_1
				SRR30554020	H_2067_1
				SRR30554019	H_2068_1
				SRR30554018	H_2080_1
				SRR30554017	H_2081_1
				SRR30554016	H_2082_1
				SRR30554014	H_2083_1
				SRR30554013	H_2084_1
				SRR30554012	H_2085_1
				SRR30554011	H_2086_1
				SRR30553978	H_2087_1
				SRR30553977	H_2088_1
				SRR30553956	H_2160_1

SRR30553955	H_2161_1
SRR30553953	H_2162_1
SRR30553976	H_2163_1
SRR30553974	H_2164_1
SRR30553973	H_2165_1
SRR30553972	H_2166_1
SRR30553971	H_2167_1
SRR30553970	H_2168_1
SRR30553969	H_2180_1
SRR30553968	H_2181_1
SRR30553967	H_2182_1
SRR30553966	H_2183_1
SRR30553965	H_2184_1
SRR30553963	H_2185_1
SRR30553961	H_2186_1
SRR30553960	H_2187_1
SRR30553959	H_2188_1
SRR30553958	H_4000_1
SRR30553957	H_4001_1
SRR30553997	H_4002_1
SRR30553996	H_4003_1
SRR30553995	H_4004_1
SRR30553952	H_4005_1
SRR30553950	H_4006_1
SRR30553949	H_4007_1
SRR30553948	H_4008_1
SRR30553947	H_4010_1
SRR30554010	H_4011_1
SRR30554009	H_4012_1
SRR30554008	H_4013_1
SRR30554007	H_4014_1
SRR30554006	H_4015_1
SRR30554005	H_4016_1
SRR30553962	H_4017_1
SRR30554003	H_4018_1
SRR30554002	H_4060_1
SRR30554001	H_4061_1
SRR30554000	H_4062_1
SRR30553999	H_4063_1

SRR30553998	H_4064_1
SRR30553994	H_4065_1
SRR30553954	H_4066_1
SRR30553941	H_4067_1
SRR30553993	H_4068_1
SRR30553992	H_4080_1
SRR30553991	H_4081_1
SRR30553990	H_4082_1
SRR30553989	H_4083_1
SRR30553988	H_4084_1
SRR30553987	H_4085_1
SRR30553986	H_4086_1
SRR30553985	H_4087_1
SRR30553984	H_4088_1
SRR30553982	H_4160_1
SRR30553981	H_4161_1
SRR30553980	H_4162_1
SRR30553979	H_4163_1
SRR30553946	H_4164_1
SRR30553945	H_4165_1
SRR30553944	H_4166_1
SRR30553943	H_4167_1
SRR30553942	H_4168_1
SRR30553939	H_4180_1
SRR30554034	H_4181_1
SRR30554035	H_4182_1
SRR30554036	H_4183_1
SRR30554037	H_4184_1
SRR30554038	H_4185_1
SRR30554039	H_4186_1
SRR30554040	H_4187_1
SRR30554041	H_4188_1

Table S2. NCBI accession number list at root endosphere samples.

Bioproject	Name	Biosample	Sample	accession	library_ID
PRJNA1156826	24-FunBio-FO_Rh_SP6C4_S8-31July	SAMN43501991	Root endosphere	SRR30556097	R_2000_1
				SRR30556096	R_2001_1
				SRR30556071	R_2002_1
				SRR30556057	R_2003_1
				SRR30556068	R_2004_1
				SRR30556049	R_2005_1
				SRR30556038	R_2006_1
				SRR30556025	R_2007_1
				SRR30556013	R_2008_1
				SRR30556002	R_2010_1
				SRR30556095	R_2011_1
				SRR30556086	R_2012_1
				SRR30556085	R_2013_1
				SRR30556084	R_2014_1
				SRR30556083	R_2015_1
				SRR30556082	R_2016_1
				SRR30556081	R_2017_1
				SRR30556080	R_2018_1
				SRR30556079	R_2060_1
				SRR30556078	R_2061_1
				SRR30556072	R_2062_1
				SRR30556073	R_2063_1
				SRR30556074	R_2064_1
				SRR30556075	R_2065_1
				SRR30556076	R_2066_1
				SRR30556077	R_2067_1
				SRR30556053	R_2068_1
				SRR30556054	R_2080_1
				SRR30556055	R_2081_1
				SRR30556056	R_2082_1
				SRR30556058	R_2083_1
				SRR30556059	R_2084_1
				SRR30556060	R_2085_1
				SRR30556061	R_2086_1
				SRR30556062	R_2087_1
				SRR30556063	R_2088_1
				SRR30556064	R_2160_1

SRR30556065	R_2161_1
SRR30556066	R_2162_1
SRR30556067	R_2163_1
SRR30556069	R_2164_1
SRR30556070	R_2165_1
SRR30556052	R_2166_1
SRR30556051	R_2167_1
SRR30556033	R_2168_1
SRR30556032	R_2180_1
SRR30556031	R_2181_1
SRR30556030	R_2182_1
SRR30556029	R_2183_1
SRR30556050	R_2184_1
SRR30556048	R_2185_1
SRR30556047	R_2186_1
SRR30556046	R_2187_1
SRR30556045	R_2188_1
SRR30556044	R_4000_1
SRR30556043	R_4001_1
SRR30556042	R_4002_1
SRR30556041	R_4003_1
SRR30556040	R_4004_1
SRR30556039	R_4005_1
SRR30556037	R_4006_1
SRR30556036	R_4007_1
SRR30556035	R_4008_1
SRR30556034	R_4010_1
SRR30556010	R_4011_1
SRR30556009	R_4012_1
SRR30556008	R_4013_1
SRR30556028	R_4014_1
SRR30556027	R_4015_1
SRR30556026	R_4016_1
SRR30556024	R_4017_1
SRR30556023	R_4018_1
SRR30556022	R_4060_1
SRR30556021	R_4061_1
SRR30556020	R_4062_1
SRR30556019	R_4063_1

SRR30556018	R_4064_1
SRR30556017	R_4065_1
SRR30556016	R_4066_1
SRR30556015	R_4067_1
SRR30556012	R_4068_1
SRR30556011	R_4080_1
SRR30556007	R_4081_1
SRR30556006	R_4082_1
SRR30555992	R_4083_1
SRR30555991	R_4084_1
SRR30555990	R_4085_1
SRR30556005	R_4086_1
SRR30556004	R_4087_1
SRR30556003	R_4088_1
SRR30556001	R_4160_1
SRR30556000	R_4161_1
SRR30555999	R_4162_1
SRR30555998	R_4163_1
SRR30555997	R_4164_1
SRR30555996	R_4165_1
SRR30555995	R_4166_1
SRR30555994	R_4167_1
SRR30555993	R_4168_1
SRR30556014	R_4180_1
SRR30556094	R_4181_1
SRR30556093	R_4182_1
SRR30556092	R_4183_1
SRR30556091	R_4184_1
SRR30556090	R_4185_1
SRR30556089	R_4186_1
SRR30556088	R_4187_1
SRR30556087	R_4188_1

Table S3. Coverage rate of microbial community.

Sample	Name	Good's coverage (%)	Chao's coverage (%)	Sample	Name	Good's coverage (%)	Chao's coverage (%)
Rhizosphere 2 week	H_2000	99.99906	99.99906	Root endosphere 2 week	R_2000	100	100
	H_2001	100	100		R_2001	100	100
	H_2002	100	100		R_2002	100	100
	H_2003	100	100		R_2003	100	100
	H_2004	100	100		R_2004	100	100
	H_2005	100	100		R_2005	100	100
	H_2006	100	100		R_2006	100	100
	H_2007	100	100		R_2007	100	100
	H_2008	99.99908	99.99908		R_2008	100	100
	H_2010	100	100		R_2010	100	100
	H_2011	100	100		R_2011	100	100
	H_2012	100	100		R_2012	100	100
	H_2013	100	100		R_2013	99.99834	99.99834
	H_2014	100	100		R_2014	100	100
	H_2015	100	100		R_2015	100	100
	H_2016	100	100		R_2016	100	100
	H_2017	100	100		R_2017	100	100
	H_2018	100	100		R_2018	100	100
	H_2060	100	100		R_2060	100	100
	H_2061	100	100		R_2061	100	100
	H_2062	100	100		R_2062	100	100
	H_2063	100	100		R_2063	100	100
	H_2064	100	100		R_2064	100	100
	H_2065	100	100		R_2065	100	100
	H_2066	100	100		R_2066	100	100
	H_2067	100	100		R_2067	100	100
	H_2068	100	100		R_2068	100	100
	H_2080	100	100		R_2080	100	100
	H_2081	100	100		R_2081	100	100
	H_2082	99.99895	99.99895		R_2082	100	100
	H_2083	100	100		R_2083	100	100
	H_2084	100	100		R_2084	100	100
	H_2085	100	100		R_2085	100	100
	H_2086	100	100		R_2086	100	100
	H_2087	100	100		R_2087	100	100
	H_2088	100	100		R_2088	100	100

Rhizosphere 4 week	H_2160	100	100	Root endosphere 4 week	R_2160	100	100
	H_2161	100	100		R_2161	100	100
	H_2162	100	100		R_2162	100	100
	H_2163	100	100		R_2163	100	100
	H_2164	100	100		R_2164	100	100
	H_2165	100	100		R_2165	100	100
	H_2166	100	100		R_2166	100	100
	H_2167	100	100		R_2167	100	100
	H_2168	100	100		R_2168	100	100
	H_2180	100	100		R_2180	100	100
	H_2181	100	100		R_2181	100	100
	H_2182	100	100		R_2182	100	100
	H_2183	100	100		R_2183	100	100
	H_2184	100	100		R_2184	100	100
	H_2185	100	100		R_2185	100	100
	H_2186	100	100		R_2186	100	100
	H_2187	100	100		R_2187	100	100
	H_2188	100	100		R_2188	100	100
	H_4000	100	100		R_4000	100	100
	H_4001	100	100		R_4001	100	100
	H_4002	100	100		R_4002	100	100
	H_4003	100	100		R_4003	100	100
	H_4004	100	100		R_4004	100	100
	H_4005	100	100		R_4005	100	100
	H_4006	100	100		R_4006	100	100
	H_4007	100	100		R_4007	100	100
	H_4008	100	100		R_4008	100	100
	H_4010	100	100		R_4010	100	100
	H_4011	100	100		R_4011	100	100
	H_4012	100	100		R_4012	100	100
	H_4013	100	100		R_4013	100	100
	H_4014	100	100		R_4014	100	100
	H_4015	100	100		R_4015	100	100
	H_4016	100	100		R_4016	100	100
	H_4017	100	100		R_4017	100	100
	H_4018	100	100		R_4018	100	100
	H_4060	100	100		R_4060	100	100
	H_4061	100	100		R_4061	100	100
	H_4062	100	100		R_4062	100	100

H_4063	100	100	R_4063	100	100
H_4064	100	100	R_4064	100	100
H_4065	100	100	R_4065	100	100
H_4066	100	100	R_4066	100	100
H_4067	100	100	R_4067	100	100
H_4068	100	100	R_4068	100	100
H_4080	99.9991	99.9991	R_4080	100	100
H_4081	100	100	R_4081	100	100
H_4082	100	100	R_4082	100	100
H_4083	100	100	R_4083	100	100
H_4084	100	100	R_4084	100	100
H_4085	100	100	R_4085	100	100
H_4086	100	100	R_4086	100	100
H_4087	100	100	R_4087	100	100
H_4088	100	100	R_4088	100	100
H_4160	100	100	R_4160	100	100
H_4161	100	100	R_4161	100	100
H_4162	100	100	R_4162	100	100
H_4163	100	100	R_4163	100	100
H_4164	100	100	R_4164	100	100
H_4165	100	100	R_4165	100	100
H_4166	100	100	R_4166	100	100
H_4167	100	100	R_4167	100	100
H_4168	100	100	R_4168	100	100
H_4180	100	100	R_4180	100	100
H_4181	100	100	R_4181	100	100
H_4182	100	100	R_4182	100	100
H_4183	100	100	R_4183	100	100
H_4184	100	100	R_4184	100	100
H_4185	100	100	R_4185	100	100
H_4186	100	100	R_4186	100	100
H_4187	100	100	R_4187	100	100
H_4188	100	100	R_4188	100	100

Table S4. PCoA pairwise adonis in 2-week rhizosphere.

pairs	Df	SumsOfSqs	F.Model	R2	*p*.value	*p*.adjusted	sig
Untreated vs *Fusarium*	1	0.940372	32.47871	0.669958	0.001	0.00125	**
Untreated vs SP6C4	1	0.1399	4.296661	0.211693	0.001	0.00125	**
Untreated vs S8	1	0.073838	58.79537	0.786083	0.001	0.00125	**
Untreated vs *Fusarium* + S8	1	1.468808	2205.144	0.992797	0.001	0.00125	**
Untreated vs *Fusarium* + SP6C4	1	0.019018	16.94851	0.514394	0.001	0.00125	**
Fusarium vs SP6C4	1	0.467371	7.628639	0.322856	0.011	0.011786	*
Fusarium vs S8	1	0.950389	31.72086	0.664717	0.001	0.00125	**
Fusarium vs *Fusarium* + S8	1	0.06931	2.35978	0.12853	0.172	0.172	
Fusarium vs *Fusarium* + SP6C4	1	0.982918	32.95369	0.673161	0.001	0.00125	**
SP6C4 vs S8	1	0.100572	2.996098	0.157722	0.001	0.00125	**
SP6C4 vs *Fusarium* + S8	1	0.874389	26.51445	0.623657	0.001	0.00125	**
SP6C4 vs *Fusarium* + SP6C4	1	0.154182	4.611541	0.223736	0.001	0.00125	**
S8 vs *Fusarium* + S8	1	1.4962	894.0065	0.982418	0.001	0.00125	**
S8 vs *Fusarium* + SP6C4	1	0.053543	25.14245	0.611107	0.002	0.002308	**
Fusarium + S8 vs *Fusarium* + SP6C4	1	1.523124	989.1502	0.984082	0.001	0.00125	**

Table S5. PCoA pairwise adonis in 2-week root endosphere.

pairs	Df	SumsOfSqs	F.Model	R2	*p*.value	*p*.adjusted	sig
Fusarium vs *Fusarium* + S8	1	0.067128	9.622292	0.375544	0.001	0.001875	**
Fusarium vs *Fusarium* + SP6C4	1	0.255845	141.523	0.898428	0.001	0.001875	**
Fusarium vs S8	1	0.027515	4.982398	0.237456	0.005	0.00625	**
Fusarium vs SP6C4	1	0.319198	42.76157	0.727713	0.001	0.001875	**
Fusarium vs Untreated	1	0.027738	9.474226	0.371914	0.001	0.001875	**
Fusarium + S8 vs *Fusarium* + SP6C4	1	0.075953	12.07112	0.430019	0.004	0.005455	**
Fusarium + S8 vs S8	1	0.025385	2.536766	0.136851	0.087	0.087	
Fusarium + S8 vs SP6C4	1	0.156777	13.1206	0.450561	0.002	0.003	**
Fusarium + S8 vs Untreated	1	0.05485	7.400057	0.316241	0.011	0.012692	*
Fusarium + SP6C4 vs S8	1	0.164949	34.09323	0.680596	0.001	0.001875	**
Fusarium + SP6C4 vs SP6C4	1	0.112622	16.60989	0.509351	0.002	0.003	**
Fusarium + SP6C4 vs Untreated	1	0.226022	100.7432	0.862947	0.001	0.001875	**
S8 vs SP6C4	1	0.244848	23.33006	0.593186	0.001	0.001875	**
S8 vs Untreated	1	0.025739	4.319977	0.212598	0.02	0.021429	*
SP6C4 vs Untreated	1	0.355934	45.05294	0.737932	0.001	0.001875	**

Table S6. PCoA pairwise adonis in 4-week rhizosphere.

pairs	Df	SumsOfSqs	F.Model	R2	*p*.value	*p*.adjusted	sig
Untreated vs *Fusarium*	1	1.171784	10677.61	0.998504	0.001	0.001071	**
Untreated vs SP6C4	1	1.168115	11604.33	0.998623	0.001	0.001071	**
Untreated vs S8	1	0.53238	479.8938	0.967735	0.001	0.001071	**
Untreated vs *Fusarium* + S8	1	0.03114	182.9467	0.919576	0.001	0.001071	**
Untreated vs *Fusarium* + SP6C4	1	0.016019	3.30852	0.17135	0.003	0.003	**
Fusarium vs SP6C4	1	0.031239	476.319	0.967501	0.001	0.001071	**
Fusarium vs S8	1	0.472125	439.4752	0.964872	0.001	0.001071	**
Fusarium vs *Fusarium* + S8	1	1.166442	8631.508	0.99815	0.001	0.001071	**
Fusarium vs *Fusarium* + SP6C4	1	1.074077	223.4596	0.933183	0.001	0.001071	**
SP6C4 vs S8	1	0.497188	466.7494	0.966857	0.001	0.001071	**
SP6C4 vs *Fusarium* + S8	1	1.173706	9310.872	0.998285	0.001	0.001071	**
SP6C4 vs *Fusarium* + SP6C4	1	1.073545	223.7717	0.93327	0.001	0.001071	**
S8 vs *Fusarium* + S8	1	0.482903	425.5525	0.963764	0.001	0.001071	**
S8 vs *Fusarium* + SP6C4	1	0.495162	85.28152	0.842024	0.001	0.001071	**
Fusarium + S8 vs *Fusarium* + SP6C4	1	0.018577	3.816792	0.192604	0.001	0.001071	**

Table S7. PCoA pairwise adonis in 4-week root endosphere.

pairs	Df	SumsOfSqs	F.Model	R2	*p*.value	*p*.adjusted	sig
Untreated vs *Fusarium*	1	0.117132	16.13366	0.50208	0.001	0.0015	**
Untreated vs SP6C4	1	0.026297	4.941865	0.23598	0.005	0.005357	**
Untreated vs S8	1	0.356359	103.6443	0.86627	0.001	0.0015	**
Untreated vs *Fusarium* + S8	1	0.013084	3.295559	0.170794	0.034	0.034	*
Untreated vs *Fusarium* + SP6C4	1	0.023962	8.521563	0.347513	0.001	0.0015	**
Fusarium vs SP6C4	1	0.087294	10.40498	0.394054	0.002	0.0025	**
Fusarium vs S8	1	0.114986	17.67184	0.524825	0.001	0.0015	**
Fusarium vs *Fusarium* + S8	1	0.127092	18.05637	0.530191	0.002	0.0025	**
Fusarium vs *Fusarium* + SP6C4	1	0.166248	28.27166	0.638595	0.001	0.0015	**
SP6C4 vs S8	1	0.318396	69.70484	0.813313	0.001	0.0015	**
SP6C4 vs *Fusarium* + S8	1	0.026824	5.259987	0.247413	0.003	0.003462	**
SP6C4 vs *Fusarium* + SP6C4	1	0.027398	6.951295	0.302872	0.001	0.0015	**
S8 vs *Fusarium* + S8	1	0.387634	120.503	0.882786	0.001	0.0015	**
S8 vs *Fusarium* + SP6C4	1	0.422864	205.4174	0.927738	0.001	0.0015	**
Fusarium + S8 vs *Fusarium* + SP6C4	1	0.02076	8.014048	0.333723	0.001	0.0015	**

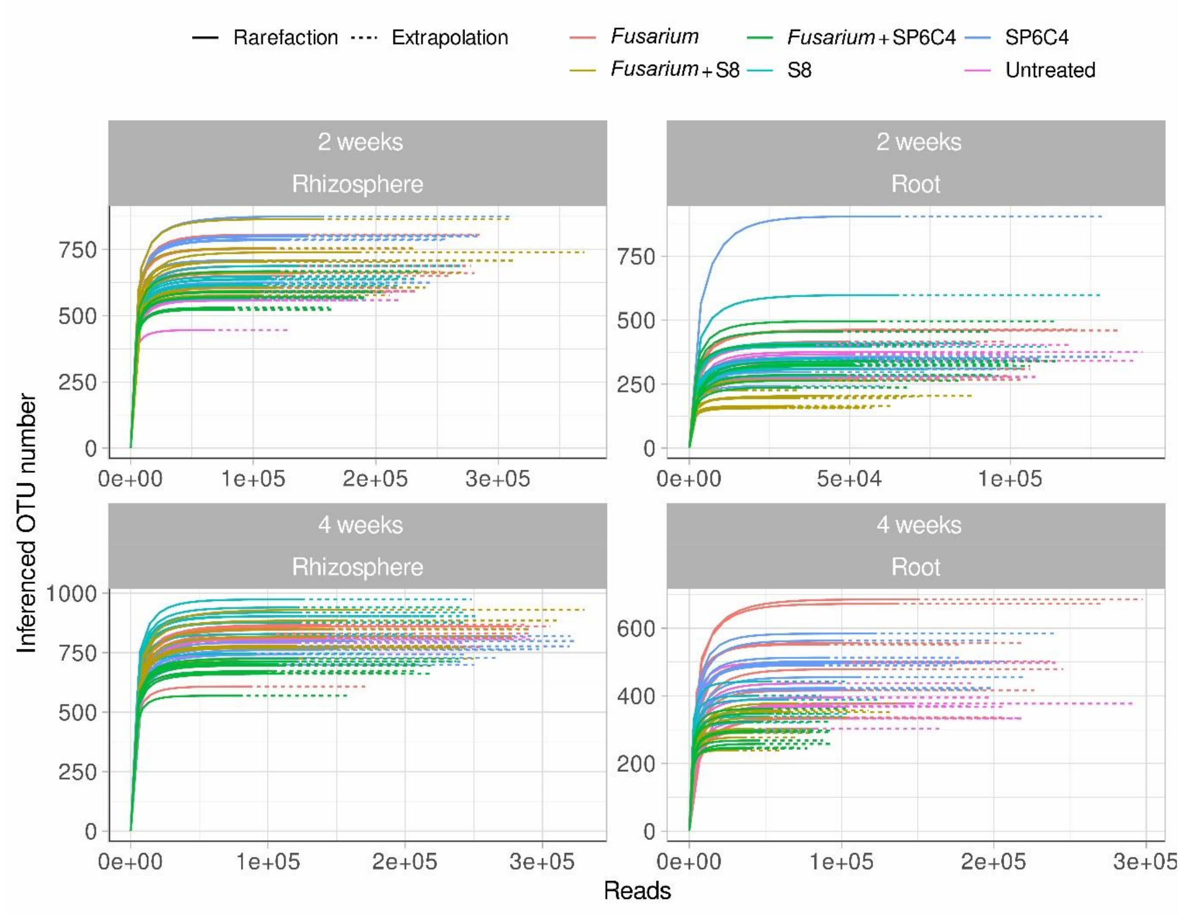

Fig S1. The rarefaction curve indicated each sequence's coverage in relation to species diversity. Rarefaction analysis was conducted using the iNEXT package in R (version 4.0.3). The solid line represents the observed sequence counts (interpolated), while the dashed line reflects the projected species diversity (extrapolated).

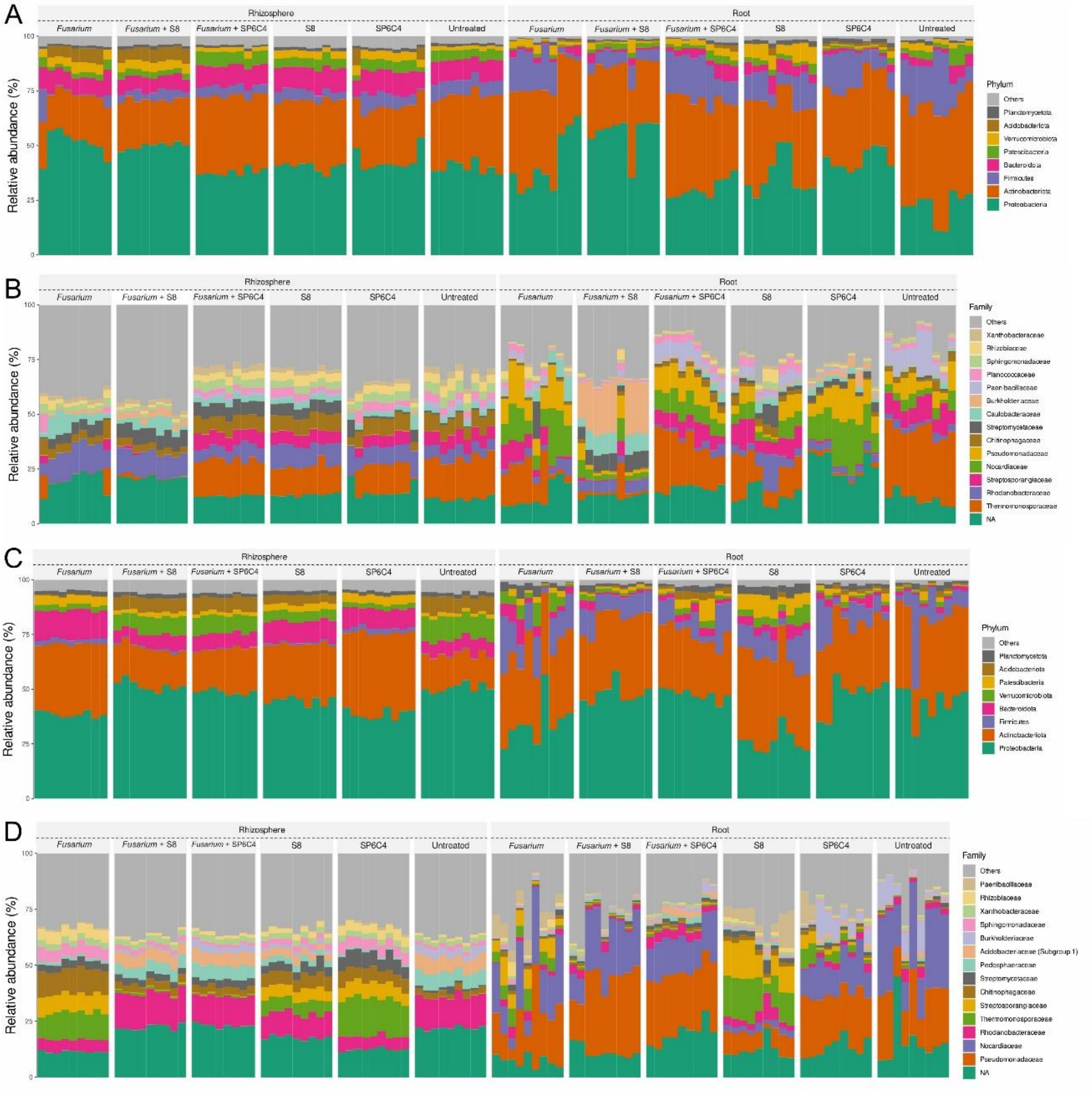

Fig S2. The bar plot elucidates the microbial community composition of the top phylum and family. (**A and B**) rhizosphere bacteria in 2 weeks. (**C and D**) rhizosphere bacteria in 4 weeks. Regarding the relative abundance of taxonomic families, the y-axis represents a relative abundance, and the color corresponds to the taxonomic group at the family level.

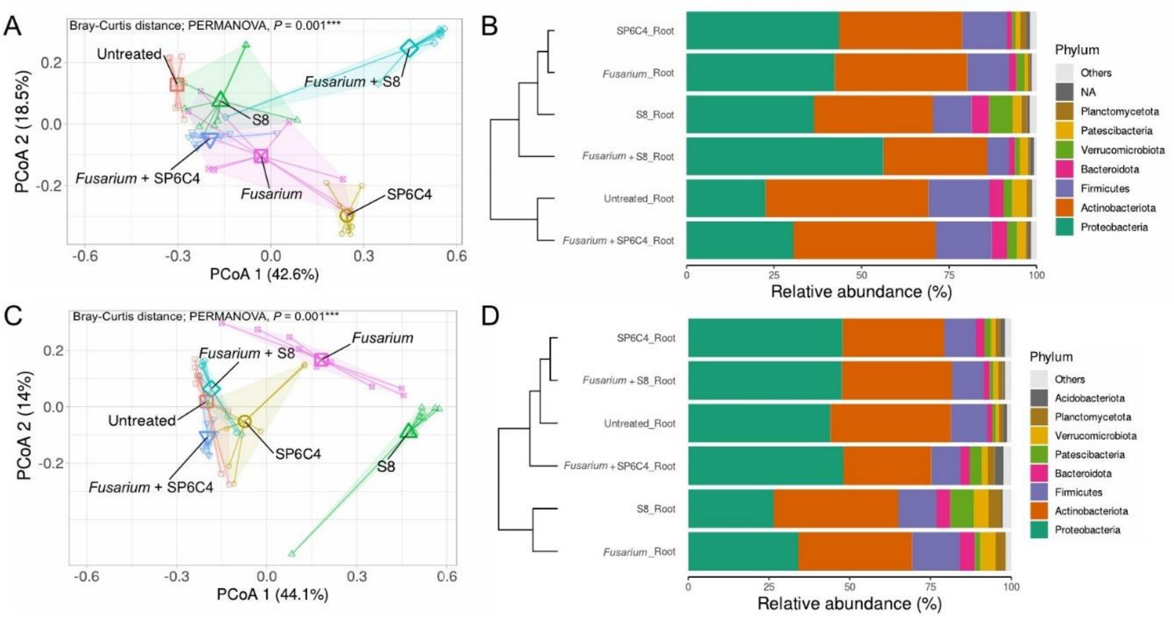

Fig S3. The composition of the rhizosphere microbial communities across all treatments in different plots is presented. (**A and C**) show the Bray-Curtis distance of ASVs based on Beta diversity analysis, visualized through classical principal coordinate analysis (PCoA). (**B and D**) display stacked bar plots representing the average percentage of bacterial populations, with microbial compositions on the right. A clustering dendrogram and hierarchical clustering, based on Bray-Curtis distance metrics, are shown on the left.
